# Scaling up interventions: findings and lessons learned from an external evaluation of Niger’s National Initiative to reduce postpartum hemorrhage

**DOI:** 10.1186/s12884-019-2502-5

**Published:** 2019-10-24

**Authors:** Meighan Mary, Ayisha Diop, Wendy R. Sheldon, Aichatou Yenikoye, Beverly Winikoff

**Affiliations:** Gynuity Health Projects, 220 East 42nd Street Suite 710, New York, NY 10017 USA

**Keywords:** Postpartum hemorrhage, Misoprostol, Uterine balloon tamponade, Non-pneumatic anti-shock garment

## Abstract

**Background:**

Niger has one of the highest maternal mortality ratios in Sub Saharan Africa, of which postpartum hemorrhage is the leading cause. In 2014, Health and Development International and the Ministry of Health of Niger launched an initiative to introduce and scale-up three PPH interventions in health facilities nationwide: misoprostol, uterine balloon tamponade, and the non-pneumatic anti-shock garment.

**Methods:**

A two-phase mixed-methods evaluation was conducted to assess implementation of the initiative. Health facility assessments, provider interviews, and household surveys were conducted in May 2016 and November 2017.

**Results:**

All evaluation facilities received misoprostol prevention doses. However, shortages in misoprostol treatment doses, UBT kits, and NASG stock were documented. Health provider training increased while knowledge of each PPH intervention varied. Near-universal uterotonic coverage for PPH prevention and treatment was achieved and sustained throughout the evaluation period. Use of UBT and NASG to manage PPH was rare and differed by health facility type. Among community deliveries, fewer than 22% of women received misoprostol at antenatal care for self-administered prophylaxis. Among those who did, almost all reported taking the drugs for PPH prevention in each phase.

**Conclusions:**

This study is the first external evaluation of a comprehensive PPH program taking misoprostol, UBT, and NASG to national scale in a low resource setting. Although gaps in service delivery were identified, results demonstrate the complexities of training, managing stock, and implementing system-wide interventions to reach women in varying contexts. The experience provides important lessons for other countries as they develop and expand evidence-based programs for PPH care.

## Background

Approximately 303,000 women died worldwide in 2015 from complications related to pregnancy and childbirth [1]. Of these deaths, 64% occurred in sub-Saharan Africa with Niger estimated to have one of the highest maternal mortality ratios in the region (553 maternal deaths per 100,000 live births). The primary cause of maternal mortality in Niger is postpartum hemorrhage (PPH), accounting for 29% of all maternal deaths [2]. Clinical protocols for childbirth in Niger require that prophylactic intra-muscular oxytocin be given to all women who deliver at health facilities. However, the need for refrigeration and administration by trained personnel poses challenges in a context, where over 84% of the population lives in rural areas and approximately 70% of deliveries occur at home, most without trained health providers [2].

Misoprostol has been established as a viable alternative to oxytocin for both prevention and treatment of PPH and has been recommended for use in low-resource settings [3, 4]. Misoprostol can be self-administered by women after delivery to help prevent PPH, and is safe to use for treatment if excessive bleeding occurs [3–5]. In addition to uterotonics, other management options for addressing PPH in low resource settings have been identified, including the uterine balloon tamponade (UBT) and non-pneumatic anti-shock garment (NASG) [6]. Health and Development International (HDI) and the Ministry of Health of Niger incorporated these technologies into a new national PPH program: the National Initiative to Fight against Postpartum Hemorrhage. The goal of the National PPH Initiative was to introduce and scale-up three new PPH interventions in health facilities: misoprostol for prophylactic use, as well as a three-step PPH treatment bundle in health facilities including misoprostol, UBT and NASG (Table 1). The aim was to scale up a package of PPH interventions nationwide at each public health facility [7]. In April 2014 the National PPH Initiative was launched. To start, a series of cascade training sessions was realized with the goal of training at least one health provider in each health facility on the new PPH protocol. Nationwide procurement was also undertaken in efforts to stock each public health facility with pre-packaged prevention doses of misoprostol (3 tablets of 200 μg each), treatment doses of misoprostol (4 tablets of 200 μg each); condom-catheter UBT kits (with the exception of health huts); and NASG.

**Table 1:** Outline of the National Initiative to Fight against Postpartum Hemorrhage

PPH prevention components:	
• Advance provision of misoprostol (600 mcg, oral) to all women at their ANC visit for use immediately after delivery during home births • Administration of misoprostol within one minute postpartum to all women delivering at health facilities, if oxytocin is unavailable	
PPH treatment bundle:	
Step 1: After PPH diagnosis, provision of misoprostol (800 mcg, sublingual) -- then, if bleeding continues for 25 min, Step 2: Insertion of uterine balloon tamponade (UBT) -- then, if bleeding continues for 6–12 min, Step 3: Placement of non-pneumatic anti-shock garment (NASG) and transfer to higher level care	
In cases of shock or uncontrolled bleeding, all three treatment interventions are to be used simultaneously	

Between May 2016 and November 2017, we conducted an external evaluation of the National PPH Initiative. The main aims of the evaluation were to:

- Evaluate training and knowledge of each PPH technology among health providers at public health facilities,
- Document availability of each new technology (misoprostol, UBT, NASG) at public health facilities, and
- Assess implementation of the National Initiative’s guidelines for PPH prevention and treatment at public health facilities;

Findings were primarily intended to inform the evolution of the national program, as well as to provide important lessons related to the feasibility of taking these interventions to scale simultaneously in low resource contexts.

## Methods

The evaluation consisted of a two-phase mixed-methods approach with repeated cross-sectional health facility assessments, including provider interviews and household surveys. The evaluation protocol was approved by the Niger Ministry of Health National Ethical Committee on March 24th, 2016. Administrative permission from the Ministry of Health was also obtained prior to implementing monitoring and evaluation activities at each public health facility in the evaluation sample. Data collection for phase 1 (May 2015–April 2016) was conducted in May 2016, followed 18 months later by phase 2 (May 2016–October 2017) in November 2017.

A two-stage purposive sampling strategy was implemented to select health facilities for the evaluation. Using population proportional to size sampling, 16 of the country’s 42 districts (2 per each of the country’s 8 geographic regions) were selected. Secondly, among the 16 evaluation districts, three primary health centers per district (48 total) were randomly selected for the study sample. In addition, given their limited number, all referral facilities in the selected districts were included in the sample. Since health huts were not included in the launch of the new initiative, they were excluded from the evaluation sample facilities. The final sample included a total of 69 health facilities: two national tertiary hospitals; seven regional tertiary hospitals; 12 district secondary hospitals; and 48 primary health centers.

### Health facility assessments

At each phase, health facility assessments were conducted at the 69 health facilities to evaluate program implementation and capacity to provide PPH prevention and treatment services according to the new PPH Initiative guidelines. Using a standardized assessment tool, trained data collectors consulted with health facility supervisors to collect information on the supply and stock management of PPH technologies, receipt of staff training on the National PPH Initiative bundle, and monthly statistics of prophylaxis and treatment of reported PPH cases. Administrative reports and medical records were also reviewed to corroborate data. At each health facility, one provider trained by the National PPH Initiative was also randomly selected for participation in an anonymous provider survey to assess supervision and training, and provider knowledge, attitudes, and practices related to the PPH Initiative.

Prior to the launch of the new initiative, health facilities systematically implemented protocols for active management for the third stage of labor using oxytocin. Use of oxytocin for PPH treatment, however, varied by provider and facility. Therefore, the health facility assessment aimed to explore use of all uterotonics for PPH management. The primary outcome for the health facility assessments was the proportion of PPH cases treated with uterotonics. Upon scale-up of the new initiative, we hypothesized an increase of 10% in the provision of uterotonic treatment for PPH cases between phase 1 and phase 2. Due to differences in the availability of oxytocin and reliability of cold chain systems, we stratified the estimated changes in uterotonic treatment by facility type and location (urban vs. rural; Additional file 1). Using an alpha = 0.05 with a two-sided test and power = 0.80, a sample size of 549 PPH cases annually was needed to test the study hypothesis. To ensure that the selected health facilities would account for the required sample size of PPH cases, we estimated PPH incidence at the selected health facilities. Based on the national institutional PPH incidence rate (1.5% [8]), we estimated that approximately 670 PPH cases would occur annually across all evaluation facilities accounting for a sufficient number of PPH cases to test the study hypothesis.

Secondary outcomes were pre-specified in the health facility assessments to address the aims of the evaluation. Some of these included:

- Proportion of health facilities with at least one trained provider
- Proportion of health facility staff trained on the PPH Initiative
- Proportion of health providers with knowledge of the misoprostol regimens, UBT insertion and removal, and placement of NASG
- Proportion of health facilities that received procurements of the new technologies
- Proportion of health facilities that experienced stock outs of the new technologies
- Facility-based implementation rates of each program component

### Household surveys

To evaluate women’s knowledge, receipt and use of advance misoprostol for PPH prevention, we also conducted household surveys with 886 recently delivered women (phase 1:455; phase 2:431). To minimize recall bias, only women who delivered within 6 months of data collection were eligible for the household survey. Household surveys were conducted by trained interviewers in all study districts except Niamey. A two-stage sampling method for recruitment of household survey participants was implemented in 14 districts. First, one primary health center was randomly selected per district for a total of 14 facilities and then within each primary health facility catchment area, villages were randomly selected. Trained interviewers approached all households within the first randomly selected village and continued until all households had been contacted; then they continued on to the next randomly selected village until sample size was obtained.

Written informed consent was obtained and the interview was conducted in local language using a standardized interview form. The interview form documented demographic characteristics and women’s receipt of PPH services. The household survey was anonymous and took approximately 45 min to complete.

For the household interviews, the primary outcome was the proportion of women that received misoprostol for PPH prevention at antenatal care (ANC). We assumed there would be an increase of 10% (from 45 to 55%) in the receipt of misoprostol for this purpose between the two phases. An alpha of 0.05, 80% power and a one-tailed test, yielded a sample requirement of 308 recently delivered women. We further assumed a 10% refusal rate and that 15% of the eligible respondents would not be available for an interview, resulting in a final sample size of 385 recently delivered women for each phase of data collection. Secondary outcomes also included the proportion of women who received information on misoprostol, took misoprostol for PPH prevention, and administered misoprostol for PPH prevention according to the PPH Initiative’s guidance.

### Statistical analyses

We compared phase 1 and phase 2 data from the health facility assessments and household surveys. We used the complex sample module in SPSS version 20 to account for the complex multistage sampling design and the clustered nature of the data. Descriptive analyses, using a Mantel-Haenszel chi-square test were conducted to assess provider training, knowledge, and stock availability of the PPH Initiative technologies. Since systematic reporting of service provision was not launched as part of the new PPH Initiative in 2014, we were unable to reliably collect data on the use of the new interventions in phase 1 in order to compare intervention utilization rates between study phases. Instead, to assess trends in implementation, we calculated average facility-based prevention and treatment rates of each program intervention by quarter from May 2016 to October 2017. During this period, service statistics were prospectively monitored and systematically documented at all health facilities in the sample. Prophylactic intervention rates were defined as the proportion of deliveries that received uterotonics while treatment intervention rates were defined as the proportion of PPH cases that received treatment using the new technologies (misoprostol, UBT, NASG). Using a paired t-test or Wilcoxon signed-rank test, we assessed differences in coverage over the 18-month evaluation period between quarter one and quarter six. *P* values ≤ 0.05 were considered statistically significant.

## Results

### Training and capacity

The number of providers trained to implement the PPH Initiative increased over the evaluation period − 90% of the sample facilities had at least one provider trained in phase 1 while all facilities had a trained provider in phase 2. By the end of the evaluation, on average, 86% of the labor and delivery staff at each facility were trained. Among providers interviewed, over 85% reported that the training on the PPH Initiative adequately prepared them for service provision and over 98% thought the new initiative was effective in improving maternal health outcomes.

Trained providers’ knowledge of the PPH technologies introduced in the National PPH Initiative varied between the evaluation phases, albeit not significantly (Table 2). Overall, knowledge of the misoprostol prevention regimen increased between phases and knowledge of how to administer the components of the PPH treatment bundle decreased. While not statistically significant, a stark decrease in knowledge of when to insert and remove UBT from 46% in phase 1 to 27% in phase 2 is noteworthy. This may be contributed to its complexity and providers’ rare use of the intervention.

**Table 2:** Provider knowledge of PPH Initiative components: % (95% CI)

	Phase 1 *N* = 60	Phase 2 *N* = 69	*p*-value
*PPH Prevention*			
Misoprostol regimen (when, dose, route)	69.9 (42.9, 87.8)	79.8 (63.8, 89.9)	*p* = .41
*PPH Treatment*			
Misoprostol regimen (when, dose, route)	78.1 (55.6, 91.0)	75.7 (54.2, 89.1)	*P* = .83
UBT insertion and removal (when)	46.3 (27.4, 66.2)	27 (14.1, 45.3)	*p* = .13
Placement of NASG	87.2 (64.7, 96.2)	78 (61.5, 88.7)	*p* = .37

Almost two times as many providers (77%) reported receiving at least one supervision visit from the Ministry of Health in phase 2 compared to phase 1 (*p* = .003). However, despite improved efforts by the Ministry of Health, the proportion of providers requesting programmatic support significantly increased by the end of the evaluation (phase 1:73%; phase 2: 95%; *p* = .005). The most frequent requests for support included the training of more providers (46.7%) and opportunities for refresher training to improve competencies (38.6%).

### Facility-based PPH care

#### PPH prevention

**Fig. 1:**
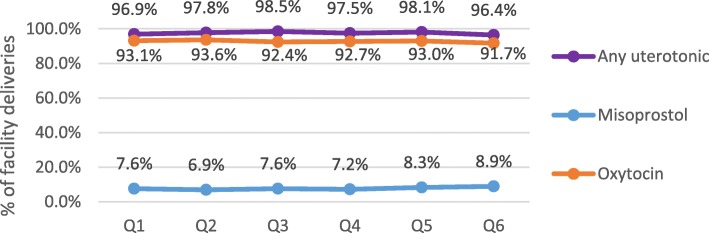
Average facility-based prophylactic coverage rates: Uterotonic administration (*N* = 69)

A total of 90,964 deliveries were documented at the study facilities over the 18-month evaluation period. Among these deliveries, near-universal prophylactic uterotonic coverage was achieved and remained stable (Fig. 1). However, due to widespread availability and use of oxytocin prophylaxis, misoprostol was administered to less than 10% of deliveries in each quarter. When stratified by facility type, prophylactic misoprostol was more frequently administered in primary health facilities. However, for each health facility level, average coverage rates for prophylactic misoprostol remained consistent across quarters with no significant increase in use from Q1 to Q6 of the evaluation period.

To assess the capacity to provide PPH prevention services, the availability of misoprostol was evaluated at each health facility (Table 3). Although not universal, facility-based availability of misoprostol prevention doses was extremely high in both phases of the evaluation. Some facilities did report experiencing a stock out within the previous 12 months. However, in most cases, the duration of the stock out was less than one month.

**Table 3:** Availability of misoprostol prevention doses: % (95% CI)

	Phase 1 (*n* = 60)	Phase 2 (*n* = 60)	*p*-value
Received at least 1 procurement	96.5 (92.3, 98.5)	98.2 (96.3, 99.2)	*P* = .23
Experienced stock out within the last 12 months	23.5 (10.0, 45.9)	28.7 (16.8, 44.5)	*p* = .64
Stock out duration within last 12 months			
Never	76.5 (54.1, 90.0)	72.7 (56.4, 84.5)	*p* = .68
Less than 1 month	12.9 (4.6, 31.1)	19.8 (9.5, 36.7)	
1–3 months	9.2 (2.4, 29.1)	7.5 (3.1, 17.3)	
Over 3 months	1.4 (0.2, 10.2)	0	
Expired stock at time of data collection	18 (7.3, 38.1)	20.4 (9.6, 38.4)	*P* = .81

#### PPH treatment

**Fig. 2:**
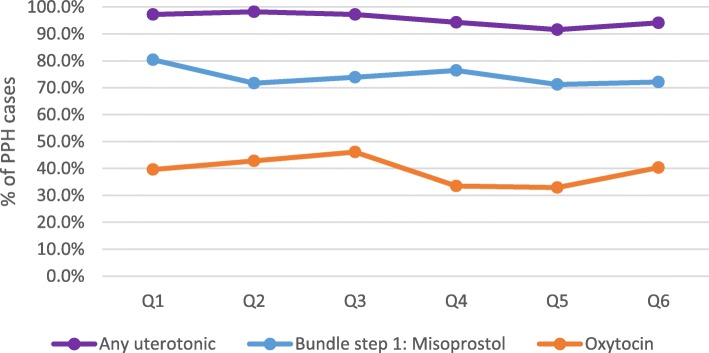
Average facility-based PPH treatment rates: Uterotonic administration (*N* = 69)

**Fig. 3:**
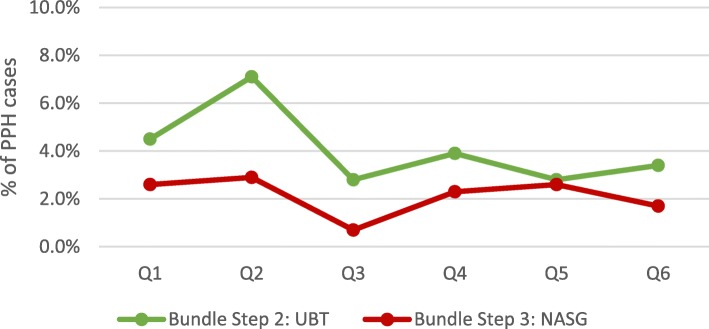
Average facility-based PPH treatment rates: UBT & NASG (*N* = 69)

**Fig. 4:**
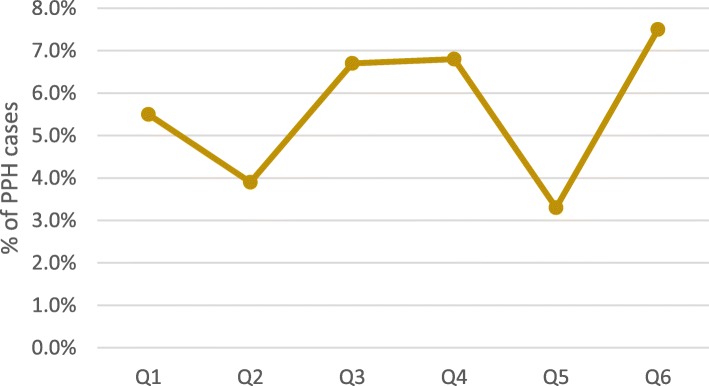
Average facility-based PPH case fatality rates (*N* = 69)

Among all documented facility-based deliveries, 1765 women were diagnosed with PPH and another 660 were referred in for emergency PPH care. Average quarterly PPH case incidence at facilities remained stable at approximately 2% of deliveries between quarter 1 and 6 (*p* = .771). Scale-up and implementation of each step of the PPH treatment bundle varied among these cases (Figs. 2 and 3). Variability in the reported facility-based PPH case fatality rate was also documented (Fig. 4) with a reported high of 7% in Q6. Yet, no significant differences were documented over the 18-month period (*p* = .213).

Implementation of step 1 of the PPH treatment bundle (misoprostol) did not reach national-scale due to lack of drug availability; by phase 2 of the evaluation, only 75% of health facilities had received misoprostol treatment doses for service provision (Table 4). Stock outs of misoprostol treatment doses were also more prevalent increasing from 25 to 53% of facilities between the two evaluation phases (*p* = .06). Reported rates of uterotonic administration for PPH treatment at health facilities (including misoprostol for step 1) was consistently high (Q1: 97%; Q6: 94%; Fig. 2). Misoprostol was predominantly used for PPH treatment despite availability of oxytocin (Q1: 80%; Q6: 72%). Average reported rates of oxytocin administration (40% in Q1 and Q6) suggest that many women received multiple uterotonic drugs for PPH treatment.

**Table 4:** Availability of interventions for PPH treatment bundle: % (95% CI)

	Phase 1 (*n* = 69)	Phase 2 (*n* = 69)	*p*-value
*Step 1: Misoprostol – treatment doses*			
Received at least 1 procurement of misoprostol treatment doses	72.8 (55.5, 91.2)	75.3 (52.1, 89.5)	*P* = .78
Experienced stock out within the last 12 months	24.7 (9.4, 50.8)	53.4 (33.1, 72.6)	*P* = .06
Stock-out levels within last 12 months			
Never	75.3 (49.2, 90.6)	47.2 (27.7, 67.7)	*P* = .07
Less than 1 month	5.4 (1.5, 17.2)	26.6 (12.0, 49.2)	
1–3 months	10.4 (2.6, 33.1)	26.1 (10.6, 51.3)	
Over 3 months	8.9 (1.2, 44.1)	0	
Expired stock at time of data collection	25.2 (11.9, 45.9)	49.8 (33.3, 66.4)	*P* = .05
*Step 2: Uterine balloon tamponade kits*			
Received at least 1 procurement of UBT kits	80 (61.5, 91.0)	80 (57.0, 92.0)	*p* = .63
Experienced stock-out within the last 12 months	0.3 (0, 2.7)	23.4 (10.1, 45.4)	*p* < .001
Stock-out levels within last 12 months			
Never	99.7 (97.3, 100)	81.8 (59.4, 93.2)	*p* = .001
Less than 1 month	0	2.1 (0.4, 9.3)	
1–3 months	0.3 (0, 2.7)	16.1 (5.3, 39.9)	
Over 3 months	0	0	
Incomplete UBT kits in stock	9.6 (3.8, 22.3)	60.1 (41.2, 76.4)	*p* < .001
*Step 3: Non-pneumatic anti-shock garment*			
Functional NASG in stock	87.9 (63.3, 96.4)	91.5 (66.7, 98.3)	*p* = .62
Quantity of NASG in stock			
None	12.1 (3.6, 33.7)	8.5 (1.7, 33.3)	*p* = .62
1–3	87.5 (66.3, 96.1)	91.1 (67.2, 98.1)	
4+	0.4 (0.2, 1.1)	0.4 (0.2, 1.0)	

UBT kits and NASGs (Step 2 & 3 of PPH treatment bundle) were not provided to all facilities by phase 2 nor did availability change between the evaluation periods (Table 4). Approximately 80% of facilities received at least one procurement of UBT kits and 92% obtained at least one NASG by the end of the evaluation. Significantly more facilities experienced stock outs of UBT kits in phase 2 of the evaluation, an increase from 0.3 to 23% (*p* = .001). Stock-outs were primarily due to the repurposing of kit’s contents (condoms, antibiotics, etc.) for other uses at the health facility rather than increased use of the UBT for PPH management. The proportion of facilities with incomplete UBT kits significantly increased from 9.6 to 60% in phase 2 (*p* < .001). In both phases, referral facilities had significantly more NASG in stock (mean: 3; range 1–10) than primary health facilities (mean: 1; range 1–3; *p* < .001). Some referral facilities had as many as 10 NASG while use of them was never reported throughout the evaluation.

**Fig. 5:**
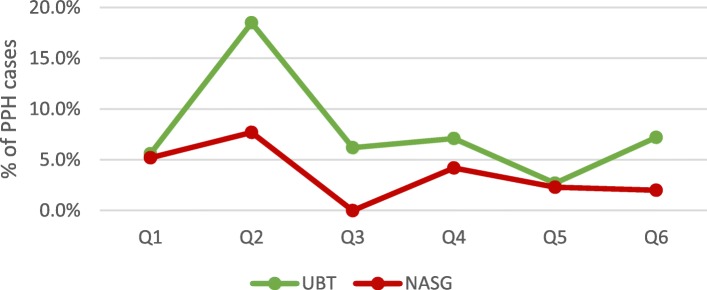
Average facility-based PPH treatment rates: UBT & NASG at primary health facilities (*N* = 48)

**Fig. 6:**
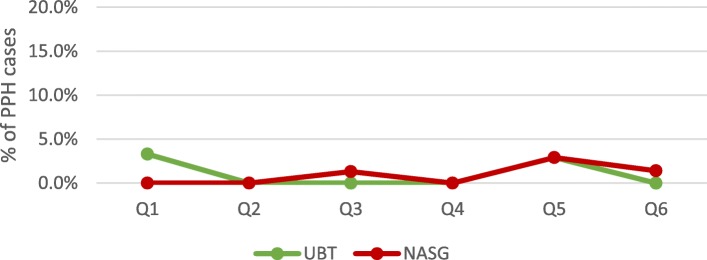
Average facility-based PPH treatment rates: UBT & NASG at referral health facilities (*N* = 21)

Despite high rates of uterotonic provision for PPH treatment, the UBT and NASG were rarely implemented for PPH management (Fig. 3). No significant changes in use were documented for any of the PPH treatment interventions over the 18 month evaluation period. Sub-analysis by health system level (primary and referral facilities) and locality (urban and rural) also did not yield any significant changes. However, there were discrepancies in quarterly UBT and NASG use between primary and referral health facilities (Figs. 5 and 6). While quarterly UBT treatment rates averaged from 3 to 18% at primary health facilities, referral facilities did not report any UBT use in four of the six quarters. Similarly, no use of NASG was reported at referral facilities in Q1, Q2, and Q4.

### Community access to preventive PPH care: household interviews

To evaluate the National PPH Initiative’s reach within communities in Niger, 886 household interviews were conducted with recently delivered women (Table 5). There were few differences in demographic characteristics of women interviewed in each phase. The vast majority of women in each phase were multiparous, with over a quarter of women reporting five or more live births. Attendance at one or more antenatal care consultation was universally high, although significantly fewer women did so in phase 2 than in phase 1 (*p* = 0.03). Less than half of women in each phase reported receiving antenatal counseling or any information on postpartum hemorrhage (phase 1: 45.8.7%; phase 2: 36.9%). A quarter of women reported receiving counseling about misoprostol for prevention of PPH (phase 1:26.3%; phase 2:24.9%). The receipt of misoprostol at ANC for self-administered prophylaxis was also low (phase 1: 22.4%; phase 2: 23.6%). Within the public health system, primary health facilities were most frequently attended for delivery. However, many women reported delivering their most recent pregnancy within their community (at home or in transit) – 32.7% in phase 1 and 47.3% in phase 2.

**Table 5:** Recently delivered women: Background and delivery characteristics: % (95% CI)

	Phase 1 (*N* = 455)	Phase 2 (*N* = 431)	*p*-value
Age in years			
≤ 20	32.4 (26.1, 39.4)	30.5 (25.2, 36.4)	*P* = .67
21–30	48.4 (40.7, 56.3)	48.3 (41.2, 55.6)	
31–40	18.4 (12.7, 25.9)	19.3 (14.8, 24.6)	
40+	0.7 (0.1, 3.5)	1.9 (0.6, 6.4)	
Highest educational attainment			
None	49.1 (36.8, 61.5)	46 (35.8, 56.7)	*P* = .77
Primary	15.8 (11.6, 21.3)	20 (12, 31.5)	
Secondary or higher	5.8 (3.3, 10)	6.6 (2.6, 15.8)	
Koranic	29.3 (17.4, 44.8)	27.4 (17.1, 40.7)	
Married	98.2 (93.7, 99.5)	96.3 (86.6, 99.1)	*P* = .40
Attended at least 1 antenatal consultation	98.7 (96.5, 99.5)	92.2 (68.8, 98.5)	*P* = .04
Received counseling/info on PPH at ANC visit	45.8 (34.3, 57.8)	36.9 (25.2, 50.5)	*p* = .36
Received counseling/info on misoprostol at ANC visit	26.3 (12.6, 46.8)	24.9 (16.5, 35.6)	p = .68
Received misoprostol at ANC visit	22.4 (10.8, 40.8)	23.6 (17.1, 31.7)	*p* = .39
Delivery location			
Tertiary	0.6 (0.1, 4.1)	0.9 (0.1, 5.6)	*P* = .48
Secondary	1.1 (0.4, 3.4)	1.0 (0.1, 7.8)	
Primary	60.2 (34.8, 81)	47.5 (30.5, 65.1)	
Health hut	5.3 (1.4, 18.3)	3.2 (0.5, 16.8)	
Private clinic	0.1 (0, 0.3)	0.1 (0, 0.3)	
Community deliveries (Home/in transit)	32.7 (15.2, 56.9)	47.3 (29.1, 66.2)	
Live births			
0	0	0.6 (0.2, 1.5)	*P* = .27
1	19.9 (17.2, 22.8)	18.8 (14, 24.8)	
2–4	50.7 (46.7, 54.8)	55.6 (47.3, 63.6)	
5+	29.4 (25.5, 33.6)	25 (19, 32.3)	

Analysis of community deliveries (Table 6) yielded similar rates in access to information about PPH and misoprostol during pregnancy. Reported distribution of prophylactic misoprostol doses at ANC decreased with 21% of women reporting having received a dose at their ANC visit in phase 1 compared to 15% in phase 2 (*p* = .37). Women who received misoprostol and delivered at home, almost always reported taking the drug (approximately 96% in both phases) with the majority reporting correct administration of the prophylactic misoprostol dose (phase 1: 94.9%, phase 2: 70%).

**Table 6:** Recently delivered women: receipt and use of prophylactic misoprostol among community deliveries % (95% CI)

	Phase 1:	Phase 2:	*p*-value
	*N* = 182	*N* = 197	
Received counseling on PPH	48.1 (28.7, 68.2)	35.3 (20.5, 53.5)	*P* = .32
Received counseling/info on misoprostol	29.6 (12.7, 54.8)	17.5 (8.1, 33.8)	*P* = .33
Received misoprostol at ANC visit	21.1 (10.8, 36.9)	15 (7.7, 27.3)	*P* = .37
	*N* = 35	*N* = 23	
Took misoprostol for PPH prevention (among those who received it)	96.5 (83.4, 99.4)	95.8 (65.4, 99.6)	*P* = .87
	*N* = 33	*N* = 22	
Took misoprostol appropriately	94.9 (73.7, 99.2)	70 (29.7, 92.8)	*P* = .07
-Correct timing	100 (100, 100)	70 (29.7, 92.8)	*P* = .25
-Correct dose	94.9 (73.7, 99.2)	86.9 (27.3, 99.1)	*P* = .46

## Discussion

Near-universal uterotonic coverage for PPH prevention and treatment at health facilities was achieved by the end of the evaluation period. Due to existing facility protocols outside the realm of the new PPH Initiative, providers primarily used oxytocin, rather than misoprostol, for PPH prevention in the provision of active management of the third stage of labor. Although the evaluation did not aim to assess the availability and use of oxytocin, near-universal uterotonic prophylactic coverage suggests that for the majority of health facilities in Niger, pre-existing protocols have been successful for PPH prevention. Meanwhile, providers chose to use misoprostol predominantly for its curative indication, despite availability of oxytocin.

Future efforts related to misoprostol for PPH management should focus on health facilities that do not have reliable access to uterotonics, namely facilities without cold chain capabilities, and/or managed by lower-level providers. Although uterotonic coverage was high, it is likely that facilities with unreliable cold chains (i.e., primary health centers and health huts) would prioritize use of misoprostol over oxytocin. Expansion of the National PPH Initiative to include health huts would equip lower-level and lay health workers who typically rely solely on referral to higher care with a means to prevent and treat PPH. Community-based studies have demonstrated that task-sharing PPH diagnosis and management with misoprostol to community health workers or birth attendants prior to referral is a safe and feasible model of care [5]. In Niger, stocking and training lay health providers to administer misoprostol for PPH management at the lowest levels of the health system could magnify the National PPH Initiative’s reach.

In a country where 70% of women deliver outside the health system at home or with untrained providers [2], provision of prophylactic misoprostol at antenatal care could result in noteworthy programmatic impact. Until skilled birth attendance is universal, community-based misoprostol distribution is a safe intervention to reduce PPH [3, 4, 9–11]. Since 2002, studies have demonstrated promising results related to the feasibility of misoprostol distribution through antenatal care visits including lower rates of bleeding, fewer PPH-related referrals, and high adherence by women to correctly administer misoprostol at home deliveries [10, 12–18]. Results from the evaluation show that while almost all women attended at least one ANC visit, only 50–60% actually delivered in a health facility. The documented low rate of advance distribution of misoprostol at ANC visits in Niger is a missed opportunity to provide women delivering at home with uterotonic prophylaxis.

Trends in advance misoprostol distribution rates combined with anecdotal evidence from provider interviews suggest that many providers were more likely to distribute misoprostol to women who are already planning facility births in hopes of preventing misuse (use of misoprostol for other purposes) and encouraging facility deliveries. Similar fears have been echoed by governments and providers in many contexts [13, 19–23]. However, evidence from this evaluation and other studies [12, 13, 15, 17, 24–26] suggests that community distribution models do not reduce facility-based delivery rates nor result in the use of misoprostol for other medical indications. In fact, documented use of misoprostol for PPH prevention within the community mirrors findings from programs in other settings [14, 22, 27] – when provided with an advance dose of misoprostol for home births, women universally use it for prophylaxis and do so appropriately.

Findings suggest that the availability of the components of the PPH treatment bundle varied over time. Challenges related to procurement documented in other low resource settings [27–29], were also evident in Niger. By the end of the evaluation, 25% of facilities had still not yet received their allocated stock of misoprostol treatment doses and among those who had, stock outs were common. Prepackaging misoprostol into separate prevention and treatment doses complicated stock management. Some facilities reported stock outs of the prepackaged prevention doses while prepackaged treatment doses in the same facility expired due to the rarity of cases. One way to improve supply management of misoprostol for treatment would be to discontinue segregated packaging of the drug for prevention versus treatment.

Since UBT and NASG are secondary steps in the PPH treatment bundle, we do not expect 100% implementation rates among reported PPH cases. It is impractical to estimate ideal implementation rates since refractory PPH (need for intervention after uterotonic failure) is quite rare [30]. Due to the rarity of these cases, providers have few opportunities to practice and maintain competencies in assembling, inserting and removing the new technologies which may have contributed to the overall low use of UBT and NASG documented during the evaluation period.

However, at referral health facilities, more PPH cases were reported and on average more providers were trained on the PPH treatment bundle increasing the likelihood that these technologies could have been used. The scarcity of UBT and NASG utilization at these facilities, therefore, suggests that rather than proficiency issues, provider reticence may have played a role. Similar to results from Dumont et al. [31], providers at referral health facilities have expressed concerns that the introduction of UBT and NASG would create an extra step in PPH care, significantly delaying surgical intervention, and ultimately contributing to worse outcomes. Differences in provider experiences with the new PPH treatment bundle underscore the complexity of behavior change and training in the scale-up of program implementation. Beyond policy change, stock procurement, and clinical training, interventions need to be acceptable to those providing the service to be successful at scale.

Given the low use of UBT and NASG by providers at referral health facilities, findings suggest that programmatic realignment may be needed for more impactful results. Since the procurement and allocation of stock in each facility was disproportionate to actual use, redistributing unused UBT kits and NASGs from referral facilities to primary health centers may be an effective strategy to increase use and the reach of the PPH Initiative. Concerted efforts should also be initiated to obtain provider buy-in at secondary and tertiary facilities. Further examination is required to identify a more targeted approach which would maximize the investment and potential benefit of the UBT and NASG within Niger’s public health system.

Lessons can be learned from the gaps in service provision and implementation issues identified in the evaluation. An iterative scale-up process with gradual implementation by district or region may be preferable. Niger is one of the first countries in Sub Saharan Africa to introduce misoprostol, UBT, and the NASG for PPH care at a national scale. The investment and buy-in of the Niger Ministry of Health and HDI are remarkable; their ambitious efforts to implement the National PPH Initiative in all health facilities nationwide in a few months period cannot be understated. However, the ability to incorporate internal monitoring and evaluation to address bottlenecks in a gradual scale-up process could have prevented some issues from being reported years after program launch. Earlier monitoring could have identified and addressed issues related to training, stock management, and provider buy-in for optimal scale-up. Yet, scaling up interventions and integrating them into existing healthcare systems is difficult for any government [32]. Spicer, et al. [33] have suggested that scaling up is largely “a craft, not a science.” Our results further reinforce this sentiment by demonstrating the complexities implementing system-wide interventions to reach women in varying contexts within one country.

One limitation of this evaluation is the possible loss of internal validity [34]. Due to the pre/post nature of the study design, the first phase of data collection may have influenced results in the second phase. Limitations related to the program’s launch, namely the lack of health facility record keeping and reporting of service statistics in the initial years of implementation, have also impacted the initial analysis plan (the assessment of implementation rates between phase 1 and phase 2). As a result, analyses evaluated quarterly implementation rates of phase 2 when ongoing monitoring and evaluation was conducted. These activities and continual inquiries related to PPH care at health facilities may have resulted in reporting bias and the plateauing effect reflected in our findings. In addition, household survey data may be subject to recall bias, since women were asked to report on care received at a birth occurring up to 6 months before the survey interview.

## Conclusions

A breadth of clinical research has been undertaken to demonstrate clinical efficacy, safety, and feasibility of misoprostol, uterine balloon tamponade and the non-pneumatic anti-shock garment for PPH management. However, an evidence base by which these clinical innovations are translated into effective large-scale programs is lacking. This study is the first external evaluation of a comprehensive PPH program attempting to take misoprostol, UBT, and NASG to national scale in a low resource setting. Although our external evaluation identified clear gaps in service delivery of the bundle of interventions in Niger’s National PPH Initiative, the results provide important lessons for other countries as they develop and expand evidence-based programs for PPH care.

## Supplementary information

**Additional file 1.** Sample size estimation to evaluate 10% change in the provision of uterotonic treatment between phase 1 and phase 2 at evaluation health facilities

## Data Availability

The datasets analyzed during the current study are available from the corresponding author upon reasonable request.

## Abbreviations

ANC: Antenatal care
HDI: Health and Development International
NASG: Non-pneumatic anti-shock garment
PPH: Postpartum hemorrhage
UBT: Uterine balloon tamponade
