# Supplementary material for: Scaling up interventions: findings and lessons learned from an external evaluation of Niger’s National Initiative to reduce postpartum hemorrhage
Source: BMC Pregnancy Childbirth. 2019 Oct 24;19:379. doi: 10.1186/s12884-019-2502-5 (PMC6814039; doi:10.1186/s12884-019-2502-5)
Supplement: Supplementary file 1 — Additional file 1. Sample size estimation to evaluate 10% change in the provision of uterotonic treatment between phase 1 and phase 2 at evaluation health facilities [file 12884_2019_2502_MOESM1_ESM.docx]

**Additional File 1:**

Title: Sample size estimation to evaluate 10% change in the provision of uterotonic treatment between phase 1 and phase 2 at evaluation health facilities

Description: Due to disparities and varying access to the public health system, we stratified the estimated changes in coverage to account for differences in facility level and location. Sample size estimation was based on PPH incidence at the selected evaluation health facilities.

| **Facility Level** | **Locality** | **# of facilities** | **Est. # of deliveries per year** | **Est. # of PPH cases per year*** | **∆ (phase 1 to phase 2) in provision of uterotonics for PPH treatment** | **Required # PPH cases per phase** |
| --- | --- | --- | --- | --- | --- | --- |
| **Secondary / Tertiary** | Urban (Niamey) | 3  (1 district;  2 regional) | 8528 | 109 | 75% to 90% | 96 |
|  | Rural** | 18  (11 district;  7 regional) | 20256 | 91 | 60% to 75% | 151 |
| **Primary** | Urban (Niamey) | 6 | 19206 | 245 | 60% to75% | 151 |
|  | Rural | 42 | 50010 | 225 | 60% to 75% | 151 |
| **Total** |  | **69** | **98000** | **670** |  | **549** |
| * These estimates were based on the following assumptions: 30% of births occur in facilities in rural areas, 85% of births occur in facilities in urban areas, 1.5% of deliveries result in PPH.  ** Data is missing for 8 rural secondary/referral health facilities | | | | | | |
